# Comparison of tracheal tube cuff pressure with two techniques: fixed volume versus pilot balloon palpation

**DOI:** 10.15171/jcvtr.2017.34

**Published:** 2017-10-13

**Authors:** Farzad Rahmani, Hassan Soleimanpour, Ali Zeynali, Ata Mahmoodpoor, Kavous Shahsavari Nia, Jafar Rahimi Panahi, Sarvin Sanaei, Maryam Soleimanpour, Robab Mehdizadeh Esfanjani

**Affiliations:** ^1^Road Traffic Injury Research Center, Tabriz University of Medical Sciences, Tabriz, Iran; ^2^Students’ Research Committee, Tabriz University of Medical Sciences, Tabriz, Iran; ^3^Anesthesiology Research Team, Department of Anesthesiology, Tabriz University of Medical Sciences, Tabriz, Iran; ^4^Emergency Medicine Research Team, Tabriz University of Medical Sciences, Tabriz, Iran; ^5^Tuberculosis and Lung Research Center, Tabriz University of Medical Sciences, Tabriz, Iran; ^6^Social Determinants of Health Research Center, Tabriz University of Medical Sciences, Tabriz, Iran; ^7^Neurosciences Research Center, Tabriz University of Medical Sciences, Tabriz, Iran

**Keywords:** Endotracheal Intubation, Tracheal Tube Cuff Pressure, Emergency Department

## Abstract

***Introduction:*** Filling tracheal tube cuff (TTC) after intubation is necessary to provide a safe airway in intubated patients. On the other hand, excessive increase or decrease in the pressure of TTC’s balloon leads into the dangerous complications such as necrosis and/or aspiration. Accordingly, in the present study, we tried to evaluate the most two common fixed volume and pilot balloon palpitation methods to control TTC pressure.

***Methods: *** In a prospective cross-sectional study that was carried out in the emergency department of Tabriz Imam Reza hospital upon 194 patients who needed intubation and from April 2015 to June 2016. The patients were randomly allocated into two equal groups. For the first the Pilot Balloon Palpation technique and for the second group 10 cc fixed volume cuff filling technique was assigned. After that, the pressure was checked with manometer and data were analyzed using SPSS software.

***Results:*** TTC pressure average in fixed volume group was 44.96±21.77 cmH2O and for palpation group, it was 118.15±22.15 cmH2O. There was a meaningful difference between two groups in terms of cuff inside pressure (*P * value <0.001) and it was meaningfully lower in fixed volume group than the first one.

***Conclusion:*** The present study showed that pilot balloon palpation or fixed volume method was not appropriate methods to assess cuff pressure during intubation and the cuff pressure must be controlled by the manometer.

## Introduction

Intubation with endotracheal tube cuff (TTC) has been regarded as a benchmark of maintaining the airway among emergency patients.^1-5^ To prevent air leakage, the cuff at the end of tracheal tube is usually filled with air during anesthesia.^6^ Another important merit of the cuff is to prevent pharyngeal contents to pass into an unconscious patient’s lungs.^7^ The standard cuff pressure to prevent air leakage and aspiration has been demonstrated to be 20-30 cm of water. The minimum leak technique and also pilot balloon technique are frequently utilized by specialists to maintain cuff pressure at its normal level.^6^

After necessary training, pilot balloon palpation has been introduced by medical staff as an alternative way to control cuff pressure duly to the facility of its procedure that could prevent the complications of excessive cuff pressure.^8^

In the emergency situation, it is common that cuff is filled with excessive air which in turn causes complications such as tracheal pain, vocal cord paralysis, tracheal stenosis, tracheoesophageal fistula formation and even tracheal perforation.^2^ Sore throat in patients after anesthesia usually comes from ischemia in oropharyngeal and tracheal mucosa.^6^

Long-term complications of excessive cuff pressure have been clearly seen in chronic conditions among patients under mechanical ventilation but in acute conditions, the complications are less clear. Although, more recent studies have demonstrated that mucosal injuries by excessive cuff pressure usually happens 1-3 hours after intubation but even in patients with unstable hemodynamic and low cardiac output, due to low mucosal perfusion, mucosal injury happens in low cuff pressure.^2^

In an investigation on children it was reported that changes in anatomical position of children neck can change cuff pressure, therefore we should control cuff pressure after any neck positional changes.^9^ In a research to evaluate cuff pressure among intubated patients it was cleared that among patients under mechanical ventilation, the cuff pressure decreased by time. Thus, it is necessary to monitor cuff pressure in order to reduce complications. According to the obtained results, cuff pressure less than 20cm of water can increase the risk of ventilation associated pneumonia.^10^

TTC pressure control was used in other situations. Goentzel et al reported that using fixed volume method to fill cuff in intubated females, the cuff pressure increases rapidly but it is lower in the case of esophageal intubation, however, this discrepancy was not observed among males.^11^ In another study about TTC pressure, they demonstrated that cuff pressure must be controlled by manometer to prevent tracheal injury.^12^

## Objective

Therefore, noting the different techniques of controlling cuff pressure after intubation, the aim of present study designated to compare TTC pressure by pilot balloon palpation method and fixed volume method with manometer.

## Materials and Methods

The study was in a type of prospective cross-sectional one that carried out in the emergency department of Tabriz Imam Reza hospital. Totally, 194 patients who needed tracheal intubation from April 2015 to June 2016 participated in the study. Regarding previous investigations^11,13^ the sample size was calculated by comparison of mean cuff pressure in both techniques (fixed volume was 69±37 and palpation was 44.5±13.07). Considering the maximum mean difference between the groups µ1-µ2 =10, study power of 80%, and confidence interval of 95%, it was calculated to be 94 patients for each group using and equations. The inclusion criterion was the patient who needed intubation while the exclusion criteria were having age less than 18 years, laryngeal problems like laryngeal stenosis, tracheal bleeding, and patients or their families’ unwillingness to participate in research. Patients’ close relatives filled consensus form before participation.

The subjects were randomly allocated into two groups using Random Allocation Software in one block model and two groups. Then, one group was assigned to use pilot balloon palpation technique while the other used 10 cc fixed volume technique to fill the cuff. In pilot balloon palpation technique, the pressure estimated based on the relative pressure of pilot balloon between two fingers (at the firmness of relaxed hypothenar region of palm) while in fixed volume technique the cuff was filled with 10 cc of air. Both techniques were carried out by the single person and he is the academic member of the university. In each group, the tracheal tube was provided from Exelint Company and it was No: 8 for adult men and No: 7.5 for adult women. All samples were checked and controlled by Mallinckrodt manometer and were recorded in researcher’s checklist. We reduced cuff pressure after using manometer, when the cuff pressure was higher than normal.

The obtained data were analyzed by SPSS software (17.0.1, SPSS Inc, Chicago). In order to describe data we used descriptive statistic methods (frequency, percentage, Mean± SD). And to show data distribution normality we utilized Kolmogorov-Smirnov test. We also applied Chi-square test to compare the qualitative variables and independent sample’s t test to compare quantitative variables. P value< 0.05 was considered to be meaningful.

## Results

The total number of participants was 194. Age average in fixed volume technique group was 63.11±21.65 years and in pilot balloon palpation it was 66.42±18.66. Table 1 shows the demographic features and patients’ vital signs in both groups. Regarding intubation 177(91.2%) was intubated using rapid sequence intubation (RSI) while 17 (8.8%) patients were intubated using Crush method.

**Table 1:** Characteristics comparison of patients in both groups

**Variables**	**Technique**		**P value**
	**Fixed Volume Technique**	**Pilot Balloon Palpation Technique**	
Age (y)	63.11±21.65	66.42±18.66	0.27
Weight (kg)	69.15±14.11	69.53±17.09	0.88
Height (cm)	163.55±2.71	162.94±2.0	0.85
Heart Rate (beat per min)	85.01±27.98	86.22±29.0	0.76
Respiratory rate (per min)	13.91±3.23	13.70±3.38	0.68
SBP (mm Hg)	96.29±16.58	92.77±12.72	0.11
DBP (mm Hg)	58.91±11.99	56.87±8.97	0.17
BT (°C)	37.12±0.74	37.05±0.76	0.53

SBP: systolic blood pressure, DBP: diastolic blood pressure, BT: body temperature.

**Figure 1:**
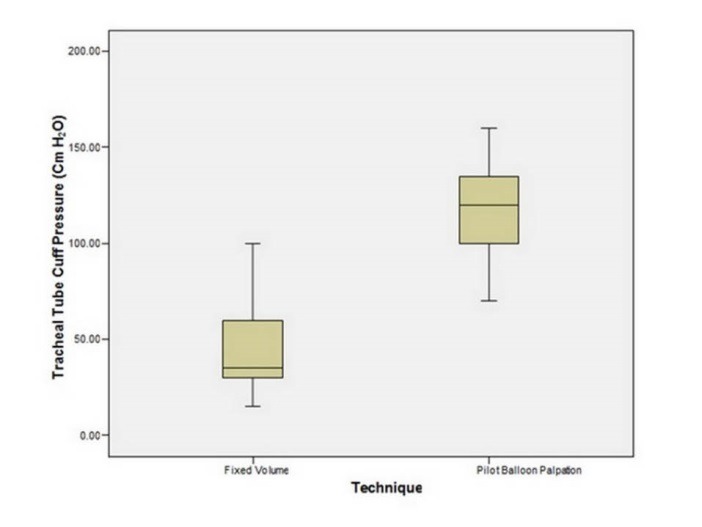
Comparison of Tracheal Tube Cuff Pressure in both group

In fixed volume group, 42 patients (43.3%) had normal cuff pressure, 9 patients (9.3%) had lower and 46 patients (47.4%) had higher cuff pressure than the normal range. While in pilot balloon palpation group, all patients had higher cuff pressure than the normal range. In fixed volume, the lowest and the highest pressure were respectively 15 cm and 100 cm of water. But in pilot balloon palpation group, they were respectively 70 cm and 160 cm of water. Figure 1 shows the distribution of TTC pressure in both groups.

The average TTC pressure of studied subjects was 44.96±21.77 cmH2O in fixed volume and it was 118.15±22.15 cmH2O in other group. There was a meaningful difference in terms of cuff pressure in two groups (*P*˂0.001). It was meaningfully lower in fixed volume compared with the palpation. By the way, both groups presented higher cuff pressure than the normal range.

## Discussion

The present study showed that cuff pressure in pilot balloon palpation group was meaningfully higher when compared with fixed volume intubation. It was significantly higher than the normal range. Moreover, by doing pilot balloon palpation technique, one cannot be completely ensured of cuff pressure and naturally one should await the inevitable consequences of high cuff pressure after intubation.

Regarding the commonality of filling cuff with the excessive amount of air by pilot balloon palpation technique in the states of emergency and as the present study suggested that it was much higher than the normal range; therefore it is better to avoid the technique.^2^ The findings of the present study are in accordance with that of other investigations. In a study by Sengupta et al to control cuff pressure in palpation technique in patients going under surgery with anesthesia they found that only in 21% of the patients the cuff pressure was measured normal. The study also revealed that in 23% of patients, it was lower than 20 cmH2O that was lower than the normal range. Finally, they suggested that to lessen the complications of inadequate cuff pressure it is necessary to control it with a manometer.^7^

In another investigation about the subject emergency patients in the hospital, they suggested in most cases the cuff pressure was higher than the normal range and caused complications.^13^ Bernon et al concluded that in order to prevent the injuries of unstable patients’ tracheal mucosa caused from high cuff pressure it must be controlled with incumbent instruments.^14^ AB Ozer et al. had a study about estimating the pressure of TTC and they concluded ineffectiveness of pilot balloon palpation to estimate cuff pressure and suggested its replacement with manometer.^15^

In another investigation it was demonstrated that pilot balloon palpation technique was not an appropriate way to control cuff pressure with over 70% higher cuff pressure than the normal range and that the pressure must be controlled by manometer.^16^ In a research by Brendt et al. they reported that to control tracheal damages, the cuff pressure must be controlled by manometer.^12^

Therefore, as mentioned, most studies suggested that in patients after intubation, the cuff pressure must be controlled by specific manometers to prevent possible complications. The findings of present investigation also agree with that of previous literature. Despite the fact that it was tried to equalize studied groups to eliminate the effect of main variables such as sex, age and patients vital sign, but in order to neutralize the effect of other confounding variables like patient’s disease, the cause of intubation, operator’s skillfulness, intubation factor and even other variables to whom the researcher had not enough knowledge was not possible. This issue can be considered as the limitation of the study.

The present investigation showed that TTC pressure during intubation was meaningfully higher in pilot balloon palpation when compared with fixed volume technique. It was significantly higher than the normal range. Therefore, pilot balloon palpation technique is not an appropriate way to control cuff pressure when compared with the fixed volume technique and manometer. Thus it is better to control the pressure with manometer.

## Competing interests

The authors declare that they have no competing interest.

## Ethical approval

The study was approved by Ethics Committee of Tabriz University of Medical Sciences and registered under the code number 5/4/10692 on February 2, 2015.

## Funding

This article was supported by Road Traffic Injury Research Center, Tabriz University of Medical Sciences, Tabriz, Iran. Special thanks to Research Vice Chancellor of Tabriz University of Medical Sciences for all the material and financial support in our study.

## Acknowledgments

The authors are grateful to all participated in the study, in addition to data collectors, supervisors, and administrative staff of Emergency Medicine, Emam Reza, Hospital, Tabriz University of Medical Sciences, Tabriz, Iran. This article was written based on dataset of Ali Zeynali’s specialty thesis entitled “Comparison of Tracheal Tube Cuff Pressure with two Techniques: Fixed Volume versus Pilot Balloon Palpation” registered in Tabriz University of Medical Sciences (No. 93/3-5/31- December 7, 2014) and was presented in September 2016.
